# Rapid Electrochemical-Based PCR-Less Microbial Quantification and Antimicrobial Susceptibility Profiling Directly From Blood and Urine With Unknown Microbial Load or Species

**DOI:** 10.3389/fbioe.2021.744198

**Published:** 2021-09-16

**Authors:** Jade Chen, Eduardo Navarro, Eliseo Nuñez, Vincent Gau

**Affiliations:** GeneFluidics, Irwindale, CA, United States

**Keywords:** direct-from-specimen, microbial quantification, electrochemical sensor, PCR-less molecular analysis, microbial responses, antimicrobial susceptibility testing, matrix interferant removal, cyclic enzymatic amplification

## Abstract

Novel molecular platforms are available for identifying (ID) the causative agents of microbial infections and generating antimicrobial susceptibility testing (AST) profiles, which can inform the suitable course of treatment. Many methods claim to perform AST in minutes or hours, often ignoring the need for time-consuming steps such as enrichment cultures and isolation of pure cultures. In clinical microbiology laboratories, an infectious microbial must first be cultured (overnight to days) and identified at the species level, followed by a subsequent AST with an additional turnaround time of 12–48 h due to the need for regrowth of the organism in the absence and presence of relevant antibiotics. Here, we present an electrochemical-based direct-from-specimen ID/AST method for reporting directly from unprocessed urine and blood in hours. In a limit of detection study of 0.5-ml whole blood samples for point-of-care and pediatric applications, 16.7% (4/24) of samples contrived at 2 CFU/ml and 100% (24/24) of samples contrived at 6 CFU/ml were reported positive in 6.5 h, indicating a limit of detection of 6 CFU/ml. In a separate direct-from-specimen AST study, the categorical susceptibility was reported correctly for blinded susceptible, intermediate, resistant, and polymicrobial contrived specimens in 4 h.

## Introduction

Any time antibiotics are prescribed to patients suspected of harboring a severe bacterial infection, the stakes are high. They are either appropriately prescribed to prevent potentially severe infections, or inappropriately prescribed, which unnecessarily contributes to the risk of adverse effects, global antibiotic resistance, and more. There is an unmet need for rapid phenotypic diagnostics in clinical settings to assist in the prescribing of antibiotics for evidence-based definitive therapy (May et al., 2013; Timbrook et al., 2017). AST technologies help accelerate the initiation of targeted antimicrobial therapy for patients with infections by inferring the concentration of an antibiotic that would be required to inhibit multiplication of a microorganism *in vitro* with acceptable toxicity for patients. Although the AST platforms that are currently most used are robust and represent added value to the clinical diagnostic microbiology laboratory, their main shortcomings are suboptimal sensitivity and specificity, a somewhat long time to result (TTR), and a lack of full automation, which may hinder the timely and accurate prescription of antibiotics (van Belkum et al., 2019). Owing to the lack of timely microbiological evidence, antibiotic therapy can only be prescribed empirically, rather than precisely upon specific target(s) (Jones and Puskarich, 2014; Han et al., 2020).

**FIGURE 1:**
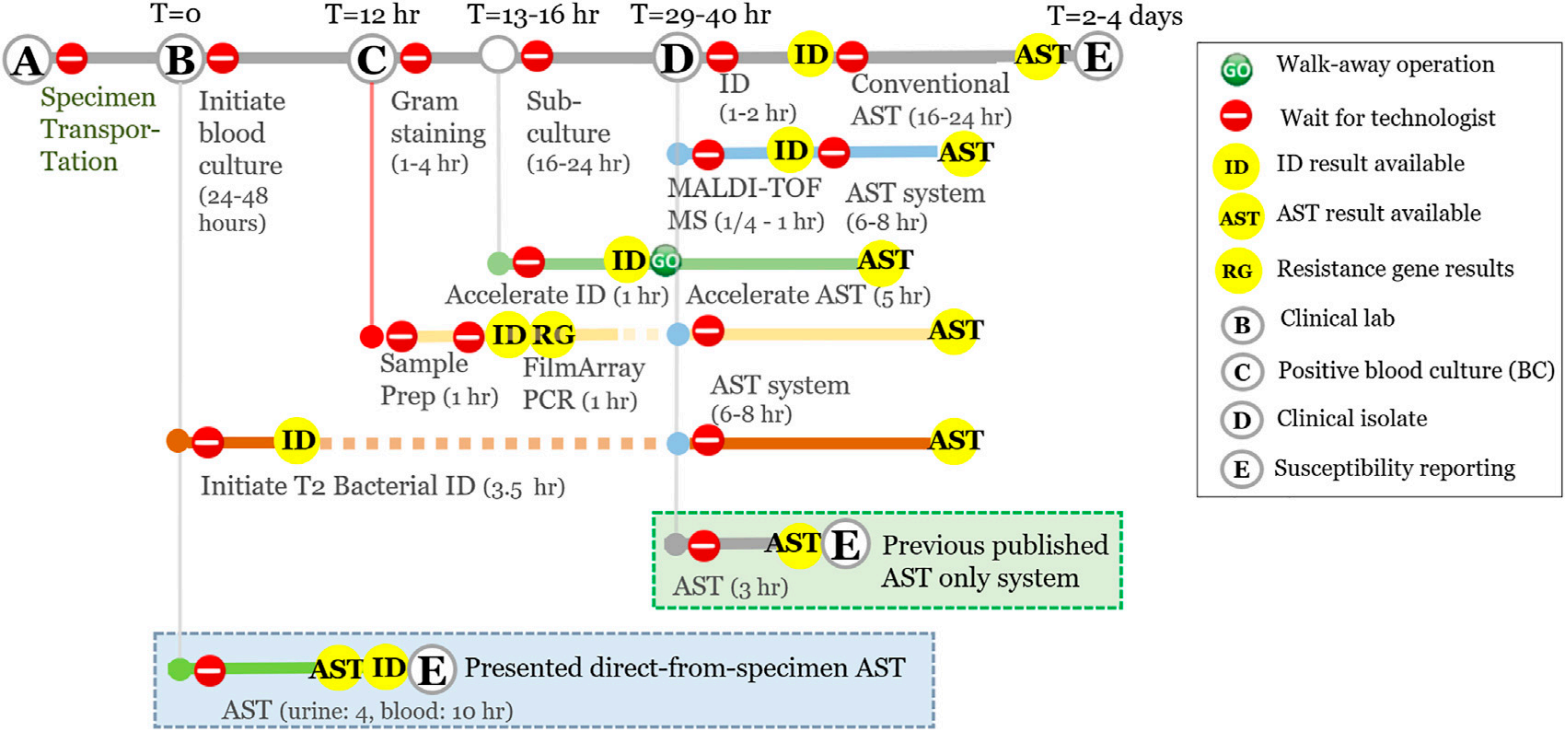
Total turnaround time of proposed direct-from-specimen assays (blue box) compared to other methods used in the clinical microbiology laboratory.

Being pivotal for the accurate management of patients with infectious diseases, identification has been significantly accelerated, miniaturized, and automated with bioMérieux VITEK2^®^, WalkAway MicroScan^®^ (Siemens), or BD Phoenix^®^ systems (Figure 1) (Anhalt and Fenselau, 1975; D’amato et al., 1981; Menozzi et al., 2006; Charretier et al., 2015; Welker, 2011). However, the selection of the right antibiotic treatment still requires time-consuming phenotypic AST according to FDA guidelines (at least 10 h following bacterial isolation), resulting in a delay of one to three days between the initiation of empirical antimicrobial therapy and the AST result (Van Belkum and Dunne, 2013). We previously published electrochemical-based microbiological response signatures that successfully distinguished resistant (R), intermediate (I), and susceptible (S) clinical isolates (not directly from specimen) using a laboratory automation system that demonstrated 98.5–100% AST categorical agreement with Clinical and Laboratory Standards Institute (CLSI) breakpoints and methodology in just 3.5 h (Figure 1 green box) (Liu et al., 2020; Chen et al., 2021a).

However, in this study, we present a direct-AST method using electrochemical sensors without any purification or nucleic acid amplification that can start directly from unprocessed specimens and test multiple samples and/or specimen types simultaneously (Figure 1 blue box). We acknowledge that most clinical microbiologists are not yet ready to accept the implementation of AST-only methods without pathogen ID, given the importance of establishing the identity of microbial species in the context of clinical decision making, as well as the potential challenges presented with performing AST directly from unprocessed clinical specimens. The first challenge derives from unprocessed polymicrobial clinical samples with multi-drug resistance. The organism with the higher bacterial load may dominate and prevent the other target(s) from being detected, or one target may be present below the limit of detection of the assay. The second challenge is the unknown microbial load of clinical specimens. According to the European Committee on Antimicrobial Susceptibility Testing and CLSI guidelines, identification of pathogens at the species level is currently an essential element in interpreting minimum inhibitory concentrations (MIC) of antibiotics for particular pathogens at a fixed inoculum concentration at 5 × 10^5^ CFU/ml (Colony Forming Unit per milliliter). The unknown microbial load from unprocessed clinical specimens is a major limitation to report susceptibility essentially equivalent to the CLSI breakpoints using CLSI methods.

**FIGURE 2:**
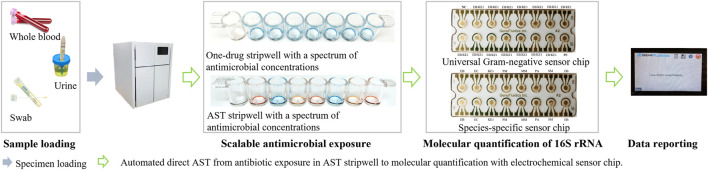
Workflow of the presented direct-AST. The user loads specimens and consumables (stripwell and sensor chip), selects specimen type, and waits for susceptibility reporting (user intervention in blue arrows). The system is designed to automate scalable antimicrobial exposure and molecular quantification (green arrows) for AST reporting directly from whole blood or urine.

To facilitate targeted antimicrobial prescribing practices and to help reduce the global burden of antibiotic resistance, the presented testing method aims to address these challenges and demonstrate categorical susceptibility reporting (R, I, S) essentially equivalent to CLSI breakpoints and methodology using contrived specimens. This electrochemical-based direct-AST method is designed for a laboratory automation system. As illustrated in Figure 2, the general operating principle of the system will consist of loading urine and blood specimens collected in their respective tube types into the system, after which the system will perform the presented AST assay directly from these unprocessed specimens. The interfering matrix components of each specimen type are removed and replaced by culture media, followed by a short 2-h antibiotic exposure using dried antibiotic stripwells consisting of different antibiotic conditions and an electrochemical sensor assay to quantify the 16S ribosomal RNA (rRNA) present in each antibiotic condition after exposure. The use of 16S rRNA as an analyte has become a common practice in many applications, such as pathogen identification, and therefore, has led to the design of a wide variety of probe pairs that detect the many characterized 16S rRNA sequences of various bacterial strains (Soriano-Lerma et al., 2020). Oligonucleotide probes complementary to target 16S rRNA sequences have been used as a tool to identify bacteria at the species level and assist in differentiating between closely related bacterial species. Chakravorty et al. suggested that utilizing a small sequence of nucleotides carrying the most taxonomic information in combination with complementary oligonucleotide probes may increase assay sensitivity and applicability to archival specimens (Chakravorty et al., 2007). The presented method has the potential to bypass a lengthy TTR and provide antibiotic susceptibility profiles in hours without the need for specimen processing or ID results.

## Methods and Material

### Cyclic Enzymatic Amplification of Chronoamperometry Measurement

The principle of the presented electrochemical-based enzymatic amplification method for molecular quantification is to convert the concentration of the target analyte such as 16S rRNA into an electrical current (*Cycling Enzymatic Amplification Using HRP and TMB*) to allow the functionalized electrochemical sensor to precisely measure the electron flow proportional to the analyte concentration (*Molecular Quantification of 16S rRNA*). The presented electrochemical sensor is a compact analytical device that incorporates a self-assembled monolayer of species or group-specific oligonucleotides as the sensing element integrated within a physiochemical transducer surface.

Chronoamperometry involves stepping the bias potential at the working electrode from an initial potential to a final potential and holding that potential while the current is recorded at the electrode. These potentials are chosen to bracket the formal potential, E_0_, of the analyte. At the initial potential, no significant current flows. Once the potential is stepped to the final potential, the analyte is consumed at the electrode surface via oxidation or reduction (depending on the direction of the step). This depletes the concentration of the analyte near the electrode. The current response is thus a rapid increase followed by a decay as the analyte is depleted and equilibrium is reached. The analysis of chronoamperometry or amperometry data is based on the Cottrell equation, which defines the current–time dependence for linear diffusion control. The final bias potential is determined such that the baseline current generated from electrolyte oxidation or reduction is minimized. This yields a better signal-to-noise ratio since the majority of background noise is from the electrolyte baseline. The Cottrell equation describes how the current, i(t)decays as a function of time, t

$$i\left(t\right)=nFAC{\left(\frac{D}{\pi }\right)}^{\frac{1}{2}}t^{-1/2}$$ (1)

where n is the number of electrons appearing in half-reaction for the redox couple, F is Faraday’s constant (96,485 C/mol), A is the electrode area (cm^2^), C is the concentration of analyte (mole/L), D is the analyte’s diffusion coefficient (cm^2^/s), *π* = 3.14159, and t is the time the current was measured in amperes. The current decays as the reciprocal of the square root of time increases. This dependence on the square root of time reflects the fact that physical diffusion is responsible for the transport of the analyte to the electrode surface. The Cottrell plot is a straight-line graph plotted as i(t) vs. $t^{-1/2}$ and can be used to determine concentration, the working electrode area, or an analyte’s diffusion coefficient.

#### Cycling Enzymatic Amplification Using HRP and TMB

**FIGURE 3:**
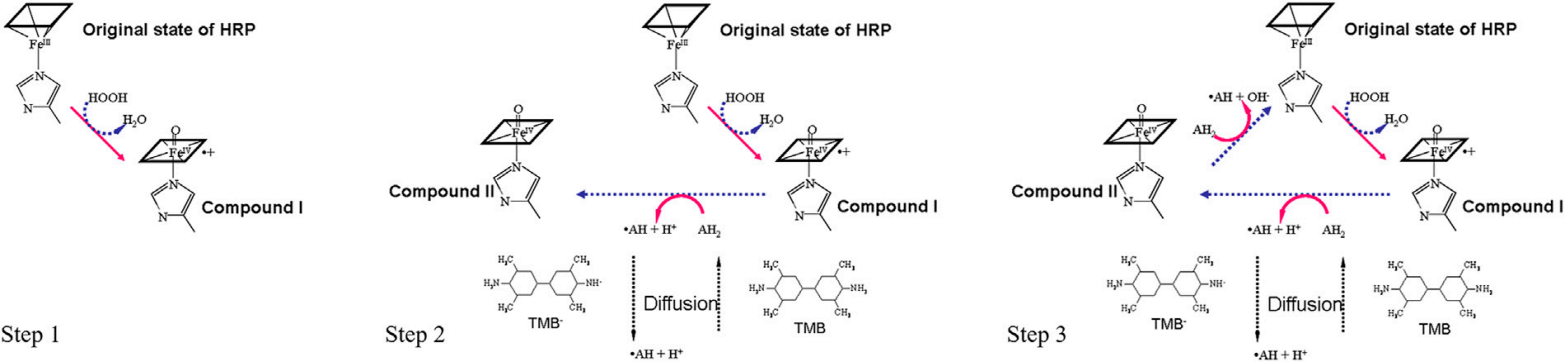
Schematic representation of the HRP-TMB cycling enzymatic amplification.

Horseradish peroxidase (HRP) is one of the most widely used enzymes for analytical purposes because its high kinetic rate maximizes enzymatic signal amplification. By converting HRP-catalyzed electron transfer to an amperometric signal, the electrochemical sensor described in this method can effectively measure the number of HRP molecules immobilized on the sensor surface. Therefore, the output current is proportional to the number of molecular targets in the sample. The generalized reaction of peroxidases is an irreversible ping-pong mechanism that can be described by three sequential steps (Figure 3):

Step 1. The H_2_O_2_ in the substrate solution will first oxidize HRP into Compound I ($Fe^{IV}=O^{-II}{\left(porphyrin\right)}^{+}$). HRP compound I is the oxidized form of HRP after losing two electrons. In other words, HRP will lose two electrons in the presence of H_2_O_2_ and will then be ready to react with TMB, the mediator.

$$Fe^{III}\left(porphyrin\right)+H_{2}O_{2}\;\overset{k_{1}}{\to }\;Fe^{IV}=O^{-II}{\left(porphyrin\right)}^{.+}+H_{2}O$$ (2)

Step 2. Oxidized HRP (Compound I) will oxidize the TMB ($AH_{2}$) in the substrate solution and turn oxidized HRP (Compound I) into Compound II ($Fe^{IV}=O^{-II}\left(porphyrin\right)H^{+}$). Concurrently, the oxidized TMB (AH) concentration will be increased and the amount will be proportional to the concentration of the analyte (16S rRNA) present. Neutral TMB is illustrated as AH_2_ and oxidized TMB is illustrated as AH in the following schematics.

$$Fe^{IV}=O^{-II}{\left(porphyrin\right)}^{.+}+AH_{2}\;\overset{k_{2}}{\to }Fe^{IV}=O^{-II}\left(porphyrin\right)H^{+}+{AH}^{.}$$ (3)

Step 3. Compound II ($Fe^{IV}=O^{-II}\left(porphyrin\right)H^{+}$) will also oxidize the TMB ($AH_{2}$) and return to the original state of HRP. This step will not only increase the concentration of oxidized TMB ($AH^{.}$) but will also bring the HRP to its original state, making it ready to go back to Step 1 and react with H_2_O_2_ again. With sufficient TMB and H_2_O_2_, HRP can continuously be “recycled” and produce abundant oxidized TMB ($AH^{.}$), which is the reactant to be measured by chronoamperometry.

$$Fe^{IV}=O^{-II}\left(porphyrin\right)H^{+}+AH_{2}\;\overset{k_{3}}{\to }\;Fe^{III}\left(porphyrin\right)+{AH}^{.}+H_{2}O$$ (4)

#### Molecular Quantification of 16S rRNA

**FIGURE 4:**
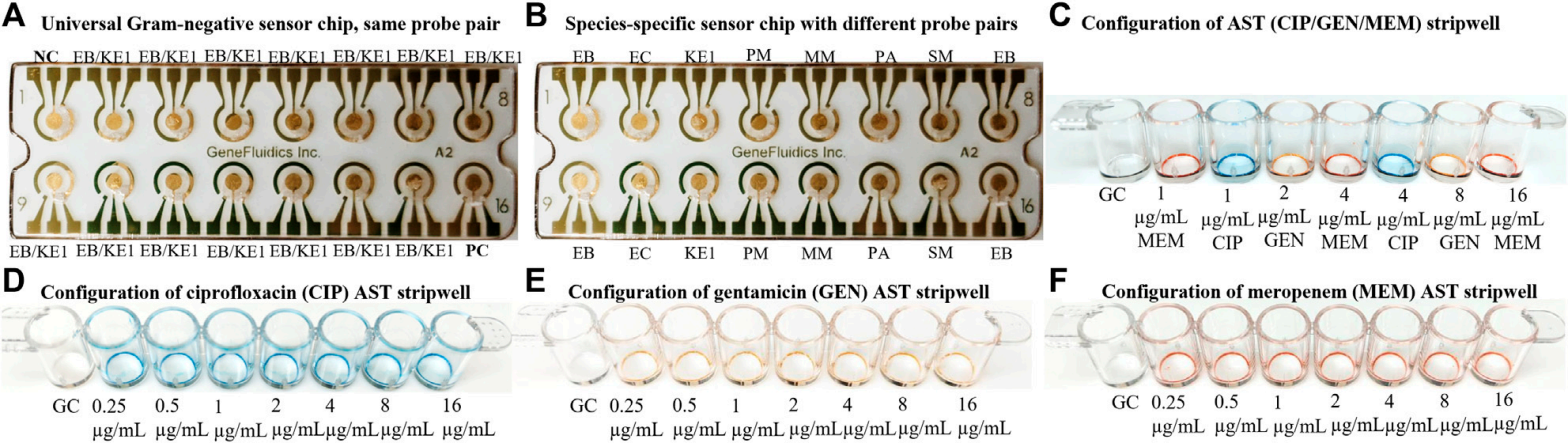
**(A)** Sensor configuration of the same probe pair (EB/KE1 for most clinically relevant Gram-negative strains including *Enterobacterales* and *Pseudomonas aeruginosa*. EB capture probe: 5′-ACT​TTA​TGA​GGT​CCG​CTT​GCT​CT-3′, EB detector probe: 5′-CGC​GAG​GTC​GCC​TTC​CTT​TGT​AT-3′, KE1 capture probe: 5′-GCA​CTT​TAT​GAG​GTC​CGC​TTG​CTC​T-3′, KE1 detector probe: 5′-CGC​GAG​GTC​GCT​TCT​CTT​TGT​ATG​C-3′) for measurement of 16S rRNA from the same species with different conditions such as calibration curve and antimicrobial responses. **(B)** Sensor configuration of different probe pairs for detection of species-specific 16S rRNA of a polymicrobial sample. NC stands for Negative Control and PC stands for Positive Control. Additional probe pairs (EC, KE1, MM, PA, SM) can be added for a customized panel for *Escherichia coli*, *Pseudomonas aeruginosa*, *Serratia marcescens*, *Morganella morganii*, *Klebsiella pneumoniae*, *Klebsiella oxytoca*, *Enterobacter cloacae*, *Klebsiella aerogenes,* and *Citrobacter freundii* to quantify 16S rRNA content individually. **(C, D)** Example of two configurations of antibiotic stripwells to be used in this study. **(E, F)** Additional configurations of antibiotic stripwells.

Electrochemical detection of 16S rRNA was performed as previously described from thiolated capture probes immobilized on photolithographically prepared gold electrode arrays with modifications (Gau et al., 2001; Sun et al., 2005; Mach et al., 2011). The detection strategy of the electrochemical-based sensors was based on sandwich hybridization of capture and detector oligonucleotide probes with target 16S rRNA in the lysate. The sensor response was evaluated with a sandwich-type hybridization assay, using FITC as a tracer in the detector probe and anti-FITC-horseradish peroxidase (HRP) as the reporter molecule. 3,3′5,5′-tetramethylbenzidine (TMB)-H_2_O_2_ was the selected substrate for the electrochemical-based cycling enzymatic amplification measurement of the activity of the captured HRP reporter. The electrochemical sensor assay provided an amperometric readout of the concentration of rRNA present in a sample. Capture and detector probe pairs were designed to hybridize to species- and group-specific regions of the 16S rRNA molecule that are accessible to independent hybridization with oligonucleotide probes on each sensor (Figure 4). The analytical performance of the multiplex electrochemical biosensor was previously investigated to demonstrate comparable signal reporting between a species-specific probe, such as the *E. coli* (EC) probe pair, and a group-specific probe, such as the *Enterobacterales* (EB) probe pair, which was a previous version of the EB/KE1 pair (Gao et al., 2017). The EB/KE1 probe pair and sensor chip configuration illustrated in Figure 4A was used for all presented experiments.

### Antibiotic Stripwells

Stripwells were prepared as previously described by drying antibiotics in DI water with 0.1% Tween onto EIA/RIA 8-well strips (Corning, Corning, NY) such that resuspension of these reagents with 100 µL of bacterial inoculum resulted in the final working concentrations for each antibiotic, or concentrations on or near the CLSI breakpoints of selected antibiotics (CLSI, 2009; Altobelli et al., 2017). The first well of each stripwell was left without antibiotic to be used as a growth control (GC). The dual-dilution antibiotic stripwell configurations used in this study are shown in Figure 4.

### Blood and Urine Sample Collection

We used BD 367884 Vacutainer Lithium Heparin tubes and BD 364954 Vacutainer^®^ Plus C&S Preservative tubes for blood and urine samples, respectively. These specimen collection tubes with the same physical dimensions were selected to allow our future lab automation system to accommodate multiple specimen types. Remnant blood and urine specimens used for preparing contrived samples were confirmed negatives. Specimens were collected under a Non-Human Subject Research determination without consent (45 CFR 46 exemption 4) under the approved New York-Presbyterian/Queens Institutional Review Board and joint master agreement.

### Bacterial Strains

Strains tested in this study included clinically relevant organisms with MIC values on or near the susceptible and resistant breakpoints of each antibiotic. Specifically, these organisms included *Enterobacter cloacae, Escherichia coli, Klebsiella aerogenes, and Klebsiella pneumoniae.* Isolates used to prepare contrived samples were obtained from the CDC AR Bank and American Type Culture Collection (ATCC).

### Whole Blood Direct Pathogen ID Assay

Two sets of 24 0.5-ml blood samples were contrived at 2 and 6 CFU/ml, after which 1.5 ml of RBC lysis buffer were added to each sample and incubated at room temperature for 9 min. Samples were then spun at 10,000 RPM for 5 min, and 1.8 ml of supernatant were removed and replaced with RBC lysis buffer. The samples were then incubated for 9 min at room temperature. Samples were spun again with the same conditions and 1.6 ml of supernatant were removed and replaced with cation-adjusted Mueller-Hinton II (MH) broth. After a 5-h viability culture at 37°C, samples were spun with the same conditions, and 1.85 ml of supernatant were removed, leaving a 150-µL pellet. To begin lysing, 90 µL of 1M NaOH were added to each sample, followed by a 5-min incubation at room temperature. Sixty microliters of 1M HCl were then added to each sample and 10 µL of lysate from each sample were added to their respective sensors on the sensor chips. All sensor chips were hybridized for 30 min at 43°C. After washing and drying each chip, 10 µL of HRP enzyme were added to all sensors, followed by a 5-min incubation at room temperature. Sensor chips were then washed and dried again, and 40 µL of TMB were added to each sensor. After a 30-s incubation at room temperature, an electrochemical reading of each sensor chip was performed.

### Matrix Removal Protocol and Inoculum Preparation for Direct-AST

Urine samples of 4-ml starting volume were spun down to remove the majority of matrix components in the supernatant as previously described (Chen et al., 2021b). After the initial 15-min spin at 4,000 RPM, 3.5 ml of supernatant were removed, followed by the addition of 3.5 ml of MH broth to the remaining 0.5-ml pellet. Urine samples were then spun again with the same conditions after which 3 ml of supernatant were removed, leaving a 1-ml volume to make a 1X inoculum. Samples were then diluted by adding 3.2 ml of MH broth to 0.2 ml of the 1X sample to create a 0.06X inoculum.

### AST From Clinical Isolates and Directly From Urine Specimens

The same AST assay was performed for clinical isolates and urine specimens with the exception of sample preparation and stripwell inoculation steps (Chen et al., 2021c). Clinical isolate suspensions were diluted to 7 × 10^6^ CFU/ml and delivered to each well of their corresponding stripwells (100 µL per well). Urine samples were prepared with the matrix removal protocol described above. Following the matrix removal and inoculum preparation steps, the 1X and 0.06X inoculum were added to their respective wells in the antibiotic stripwell. Specifically, 100 µL of 1X were added to the first four wells and 100 µL of 0.06X were added to the last four wells. The AST assay was the same for both clinical isolates and urine samples from this point. Stripwells were incubated for 2 h at 37°C for antibiotic exposure, after which the lysing procedure began. Thirty-six microliters of 1M NaOH were added to each well, followed by a 5-min incubation at room temperature. Next, 24 µL of 1M HCl were added to each well, after which 15 µL of lysed sample, or lysate, from each well were delivered to their corresponding sensors on one sensor chip (two sensors per well). All sensor chips underwent hybridization for 30 min at 43°C. Sensor chips were then washed and dried, and 10 µL of enzyme were delivered to each sensor. Following a 5-min enzyme incubation at room temperature, sensor chips were washed and dried again. Forty microliters of TMB were then added to each sensor, followed by an electrochemical reading of the sensor chip.

## Results

### Limit of Detection of Direct Whole Blood ID

**FIGURE 5:**
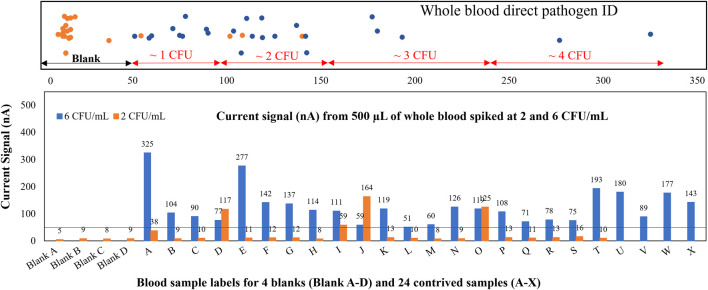
Whole blood direct pathogen ID. Gram-negative samples spiked at 2 and 6 CFU/ml, assay results reflect the probability inherent in spiking a 0.5-ml whole blood sample at approximately 2 CFU/ml.

To demonstrate the ability to detect low-abundant pathogens (<10 CFU/ml for blood) directly from limited whole blood for point-of-care applications or neonatal patients without using polymerase chain reaction (PCR) amplification, we contrived 24 whole blood samples at 2 and 6 CFU/ml of *E. coli* and took only 500 µL of blood to test on our sensors. Statistically, a large percentage of these contrived 500-µL samples should contain no pathogen at all, while some samples have 1 and very few of them have 2. As shown in Figure 5, only 4 out of 24 samples contrived at 2 CFU/ml tested positive, 1 of which indicated ∼1 CFU (sample I) and 3 of which indicated 2 CFU (samples D, J, O). These results agreed with our hypothesis. Signal ranges for each CFU count were confirmed with agar plating. Specifically, a duplicate set of all contrived samples were plated with 10 agar plates (100 µL per plate) and counted the following day. In a LoD verification experiment based on FDA guidelines, all 24 whole blood samples contrived at 6 CFU/ml tested positive. The spiked concentration was verified by plating 1 ml of the original sample on blood agar. The total assay time was 6.5 h, and the LoD was confirmed to be 6 CFU/ml with all positive signals well above the limit of blank with just 500 µL of whole blood. The electrochemical signal distribution for all samples contrived at 2 and 6 CFU/ml is shown at the top of Figure 5. The LoD can be further improved by taking larger blood volumes (2–8 ml) and/or utilizing a longer viability culture time as demonstrated previously (Chen et al., 2021d).

### Transition From Categorical AST (S, I, and R), Minimum Inhibitory Concentration (MIC), to Direct-AST Reporting

**FIGURE 6:**
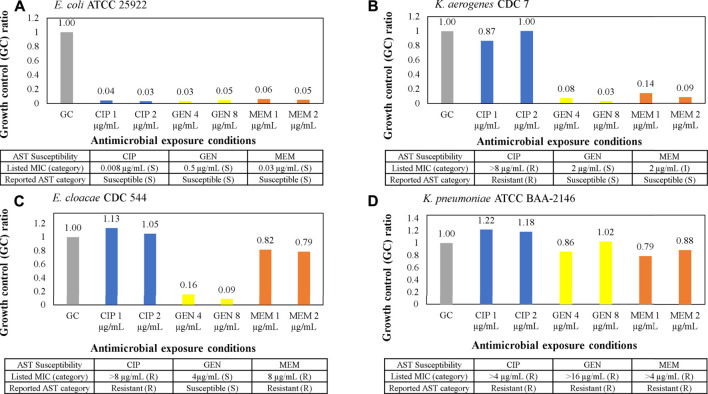
**(A–D)** AST assay from clinical isolates using the AST stripwell in Figure 4C. Microbiological response from each condition for ciprofloxacin, gentamicin, and meropenem AST stripwells with antibiotic concentrations on or near breakpoints. The microbiological response is plotted as growth control (GC) ratios, or electrochemical signal levels in nano amperes normalized to that of the GC.

Validation of microbiological responses to antibiotic exposure was performed with a panel of Gram-negative clinical isolates from the CDC AR bank and ATCC against ciprofloxacin, gentamicin, and meropenem as shown in Figure 6. Isolate suspensions were diluted to 7 × 10^6^ CFU/ml and cultured in MH broth in antibiotic stripwells. The listed MIC for each antimicrobial in the table below each graph in Figures 6A–D was verified by CLSI microdilution or published by the CDC or ATCC, and the corresponding antimicrobial susceptibility category is in parentheticals. Electrochemical signals from each antimicrobial condition were normalized to that of the GC well, resulting in a GC ratio. Low GC ratios indicated inhibited microbial growth or a susceptible response to the antibiotic conditions. High GC ratios suggested uninhibited growth or resistance to antibiotics. Our electrochemical sensor assay demonstrated categorical agreement with the listed susceptibility for all four samples against ciprofloxacin and gentamicin. However, only 3 out of 4 samples exhibited categorical agreement, and 1 sample (Figure 6B) exhibited essential agreement (only one 2-fold dilution off) against meropenem.

**FIGURE 7:**
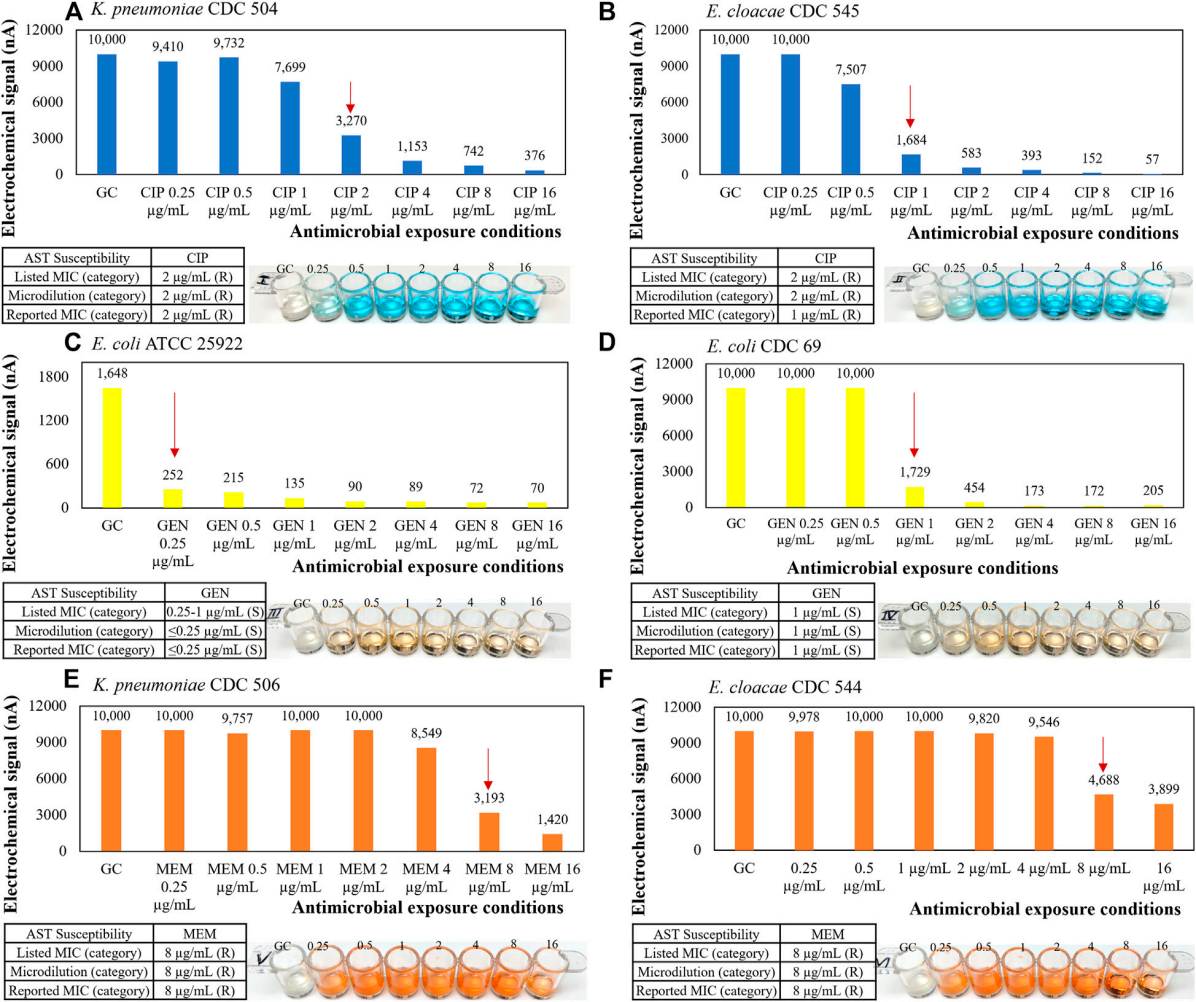
Representative AST results with microbiological response plotted in electrochemical signal levels in nano amperes for **(A, B)** ciprofloxacin, **(C, D)** gentamicin, and **(E, F)** meropenem one-drug stripwells with 3-h antimicrobial exposure and corresponding CLSI microdilution result with 16–20-h antimicrobial exposure as a reference.

The presented direct-AST protocol was evolved from a recently developed clinical specimen transportation method and molecular test based on the sequence-specific transcriptional responses of causative bacteria to antibiotic exposure directly from clinical urine specimens from NYPQ (Kung et al., 2021). The following content is from a previous publication of our stripwell protocol (Chen et al., 2021e). Figure 7 and relevant content were included in the aforementioned publication as part of the method validation results: “To verify that our AST stripwells were functionalized with the desired antibiotic concentrations, we performed the broth microdilution reference method as described in CLSI M07 (CLSI, 2009). Strains from the CDC AR Bank with different MICs for each antibiotic were suspended in Mueller-Hinton II broth and diluted to a concentration of 5 × 10^5^ CFU/ml for testing. Stripwells were inoculated with bacterial samples (100 µL per well) and incubated for 16–20 h at 37°C. After this incubation period, the reference stripwells were visually checked for microbial growth inhibition. Wells with turbid inoculum indicated microbial growth, suggesting that the antibiotic condition for that particular well was ineffective against the bacteria. Wells with clear inoculum indicated microbial growth inhibition suggesting the antibiotic condition for that well was effective against the bacteria. The concentration of the first well to exhibit complete growth inhibition was compared to the strain’s MIC as listed on the CDC AR Bank website. [Figure 7] below shows the microdilution results for AST and one-drug stripwells, as well as the results of an electrochemical biosensor assay quantifying 16S rRNA content of the inoculum in each well after the 3-h antimicrobial exposure.”

After demonstrating in Figure 7 that we can recover and detect clinically relevant microbial loads by varying the antibiotic exposure configuration, we designed the early version of our direct AST assay (Chen et al., 2021d). In this preceding version, we developed an algorithm for determining the MIC by identifying the maximum GC ratio reduction in 16S rRNA quantification over a spectrum of antimicrobial exposure conditions. Rather than using one stripwell as shown in Figures 6, 7, we used two antibiotic stripwells inoculated with clinical specimens at the original concentration (1X) and at a 10-fold dilution (0.1X) to widen the spectrum of antimicrobial exposure conditions, thus, introducing the first dual-dilution configuration to be simplified in the presented study. Urine samples of various microbial loads ranging from 10^5^ to 10^8^ CFU/ml were tested with the earlier version of the direct-AST assay, which also included a matrix removal step and a 2-h antimicrobial exposure in MH broth (Chen et al., 2021c; Chen et al., 2021d). Similarly to the AST assay performed in Figure 6, the categorical susceptibility was determined by utilizing GC ratios in a microbiological response plot against a series of 2-fold dilutions of the antibiotic. The dual-dilution kinetics corresponded to the greatest change in slope of both 1X and 0.1X response curves. The first level of analysis was qualitative, whereby the maximum inhibitory antimicrobial condition derived from the maximum GC ratio reduction was compared to the corresponding antibiotic susceptibility results (R, I, S) from the clinical microbiology lab or CLSI reference methods. The electrochemical current reading of our potentiostat reader is set to saturate at 10,000 nA to maximize the resolution at lower current readings around the limit of detection, so the reading would be saturated if the starting microbial load were too high (>10^8^ CFU/ml). This phenomenon was observed in the 10^7^ and 10^8^ CFU/ml samples. The reported inhibitory antimicrobial concentration or bug-to-drug ratio was adjusted down for every antibiotic concentration reported saturated at 10,000 nA and rounded up to the closest breakpoint, so the susceptibility reporting based on the inhibitory antimicrobial concentration of combined responses from both dilutions would be adjusted accordingly as described above.

### A Dual-Dilution Response Curve Library for Direct-From-Specimen AST

**FIGURE 8:**
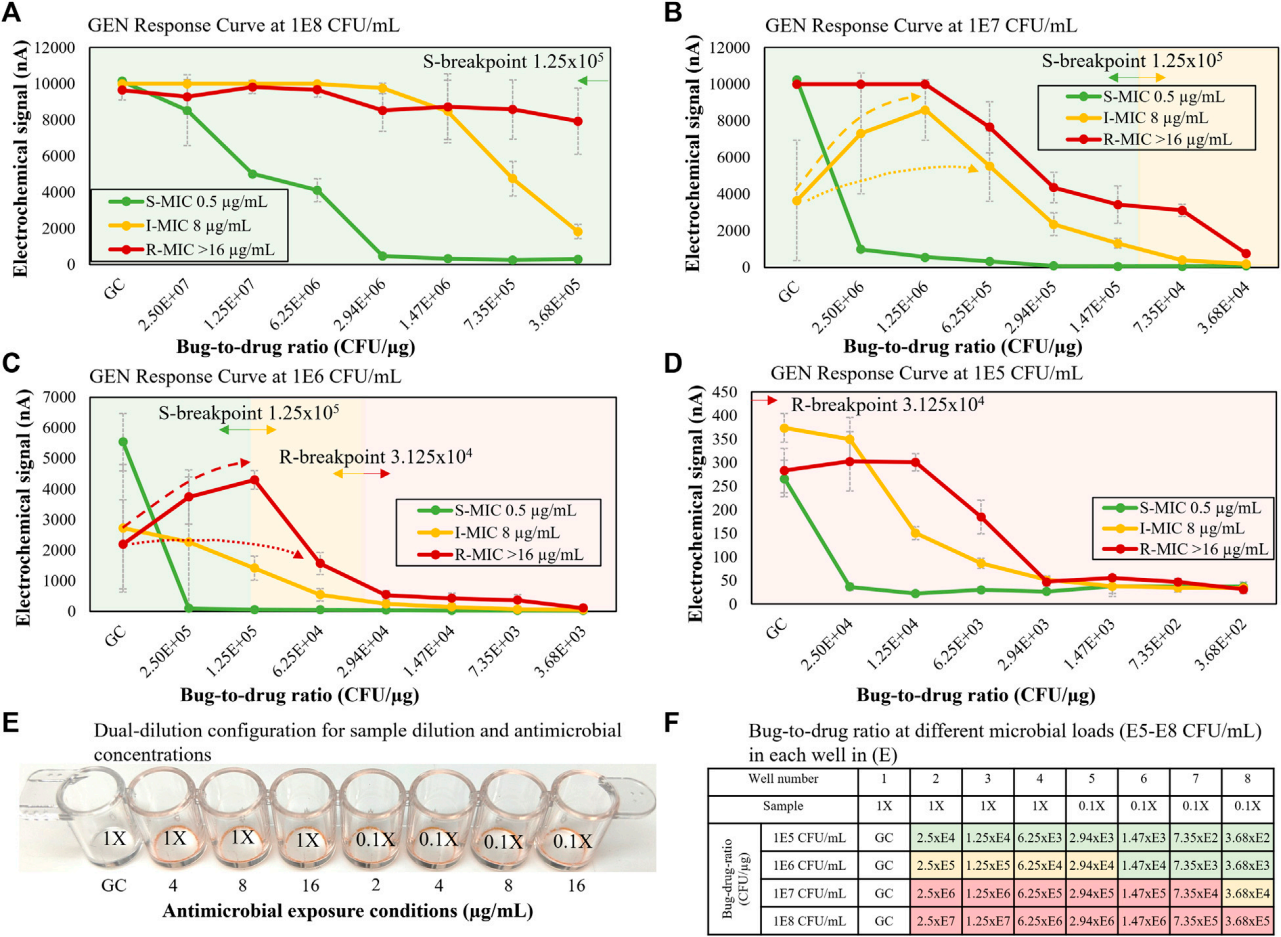
**(A–D)** Gentamicin S, I, R response curves for microbial loads of 10^5^–10^8^ CFU/ml. **(E)** Stripwell configuration used for the dual-dilution microbial response curves shown in **(A–D)**. **(F)** Bug-to-drug ratios for different microbial loads from 10^5^ to 10^8^ CFU/ml with relation to S and R breakpoints. Green cells represent bug-to-drug ratios lower than an inoculum concentration of 5 × 10^5^ CFU/ml with the S breakpoint antimicrobial concentration. Red cells represent bug-to-drug ratios higher than an inoculum concentration of 5 × 10^5^ CFU/ml with the R breakpoint antimicrobial concentration. Yellow cells represent intermediate microbial responses between S and R breakpoints.

The previous dual-dilution configuration was designed to expand the bug-to-drug ratio spectrum by using two one-drug stripwells (Figure 4E), one for 1X and one for 0.1X, to cover the clinically relevant microbial load range and CLSI R and S breakpoints defined at the reference inoculum concentration of 5 × 10^5^ CFU/ml. In the new configuration shown in Figure 8, we changed the inoculum dilution from 0.1X to 0.06X to consolidate the antimicrobial spectrum tested and use only one stripwell rather than two. A set of 3 representative strains (R, I, S) for gentamicin was used to generate a preliminary abbreviated response curve library as shown in Figure 8. The dynamic microbial range was initially set to 3 orders of magnitude (10^5^ to 10^8^) CFU/ml for urine). With the gentamicin concentrations indicated in Figure 8E and the dual-dilution of the microbial load, the corresponding bug-to-drug ratio spectrum was 2.5 × 10^7^ to 3.68 × 10^2^ CFU/µg as shown in Figure 8F, covering both reference R and S breakpoints (bug-to-drug ratios at 3.125 × 10^4^ and 1.25 × 10^5^ CFU/µg) at the high and low ends of the microbial dynamic range. As detailed in Figure 8F, the bug-to-drug ratio in wells 6, 7, and 8 are similar to those in wells 2, 3, and 4 for the microbial load that is 10 times lower in order to observe microbiological trends with similar bug-to-drug ratios at different microbial loads; we designed the spectrum as such to widen the antibiotic coverage for any unknown microbial load within the target dynamic range. For example, when looking at the I strain with a gentamicin MIC of 8 μg/ml, the signal levels from wells 6, 7, and 8 of 10^7^ CFU/ml (Figure 8B) were 1,317.59, 396.03, and 181.20 nA; those from wells 2, 3, and 4 of 10^6^ CFU/ml (Figure 8C) were 2,995.91, 1415.58, and 541.08 nA. Signal levels from wells 2, 3, and 4 in Figure 8C were higher because the inoculum concentration (1X of 10^6^ CFU/ml) was higher than that of Figure 8B (0.06X of 10^7^ CFU/ml). If each well was normalized to the GC level, the resulting GC ratios would be 0.83, 0.39, and 0.15 for Figure 8C and 0.27, 0.08, and 0.04 for Figure 8B. Although the GC ratio drop was more apparent in wells 2, 3, and 4 in Figure 8C, a similar decreasing trend was observed in both Figures 8B,C. The larger GC ratio drop observed in Figures 8B,C was due to the inoculum concentration of 10^6^ CFU/ml, which is closer to the load of 7 × 10^6^ CFU/ml used in our previous AST assay from clinical isolates designed to match CLSI breakpoints. We observed a sudden increase in rRNA synthesis caused in part by antibiotic exposure in the R strain in Figure 8B and the I strain in Figure 8C (dashed arrows) as described by the literature (Starosta et al., 2014; Vargas-Blanco and Shell, 2020). For curves exhibiting this type of response, the current algorithm calibrated the response signature (dashed lines) to the dotted lines using the GC signal as illustrated in Figures 8B,C. Figures 8A-D were used as the signature library to interpretate susceptibility of blinded samples in the next portion of the study. Bacterial strain information and GC ratios for each condition in Figure 8 may be found in Supplementary Table S1.

### Contrived Blinded Samples

**FIGURE 9:**
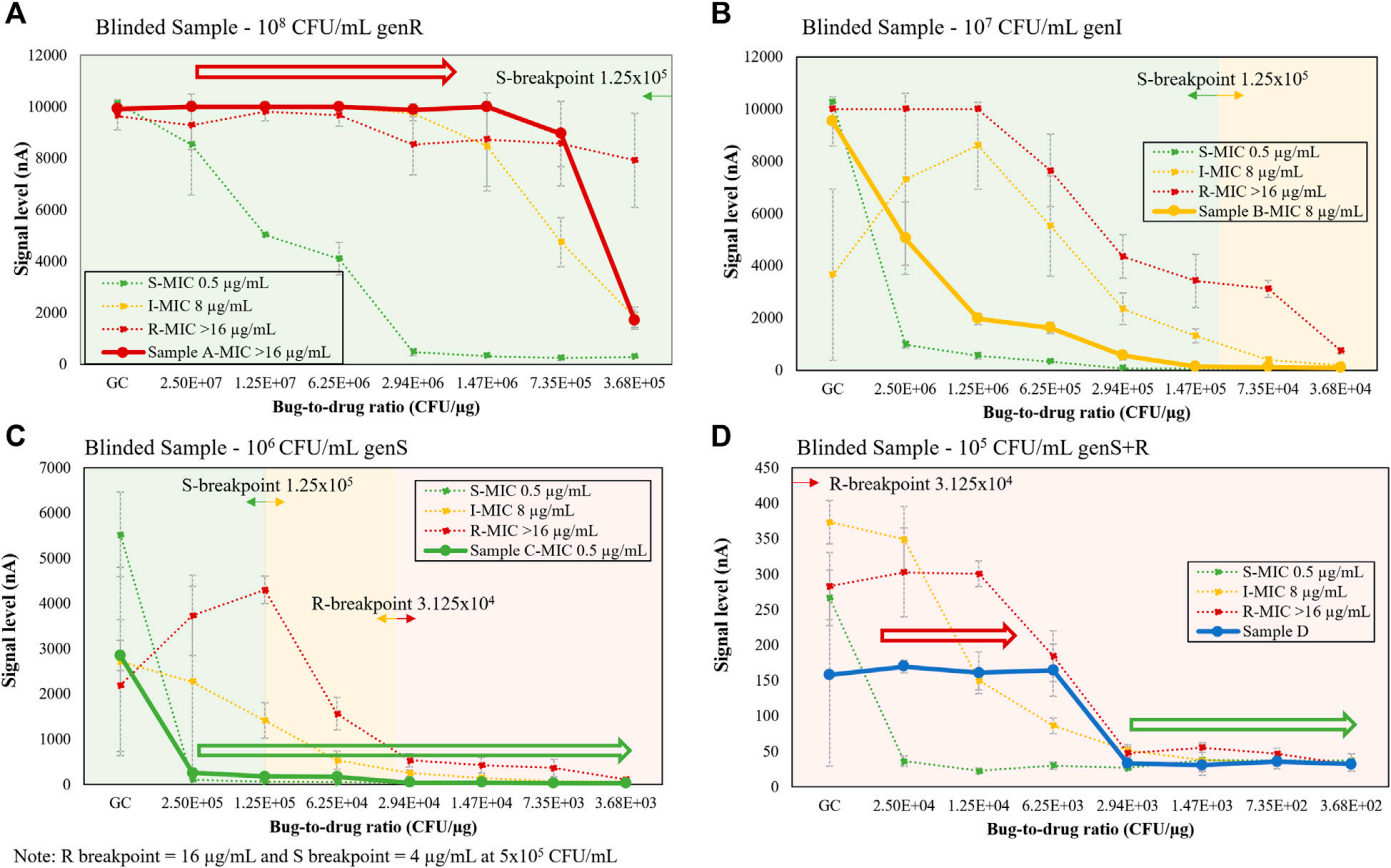
**(A–D)** Single response curve for blinded samples (solid lines), including polymicrobial, S, I, and R strains, shown on top of 10^5^-10^8^ CFU/mL signature response library (dotted lines) from Figure 8. Each strain in the polymicrobial sample was contrived at 10^5^ CFU/mL.

Negative urine samples were contrived by a different operator with 4 different microbial conditions (three monomicrobial S, I, and R strains and one polymicrobial with a different susceptibility profile), resulting in 4 blinded samples. The microbial response curves of the blinded samples using the dual-dilution configuration are shown in Figure 9. As shown below, we first estimated the microbial load of each blinded sample by comparing the GC signal level to set of previously generated calibration curves of configurable microbial quantification protocols with varying total assay times and LoDs for a range of 4 to 3 × 10^8^ CFU/ml (Chen et al., 2021d). Their resulting curves were each matched to a set of signature curves. After determining the load, we identified consecutive inhibited or non-inhibited growth trends (hollow arrows), or lack thereof, and matched the result curve to the reference curve (dotted lines) sharing the most data points or trends. Based on this analysis, all 4 blinded samples were reported correctly. Figures 9A,C exhibited non-inhibited and inhibited growth, respectively, indicating S and R susceptibility. Figure 9B did not exhibit distinct inhibited or non-inhibited growth characteristics, and the curve pattern was between I and S reference curves, indicating an intermediate response. The polymicrobial sample in Figure 9D had both inhibited (green hollow arrow) and non-inhibited growth (red hollow arrow), indicating at least one S and one R population. Bacterial strain information and GC ratios for each condition in Figure 9 may be found in Supplementary Table S2.

## Discussion

Developments in the field of rapid AST platforms have been slow over the past decade (Van Belkum and Dunne, 2013; van Belkum et al., 2019; Pulido et al., 2013), most likely due to suboptimal sensitivity and specificity and long time to result, as many clinical studies have shown that a delay in adequate antibiotic treatment for severe infections may increase mortality (Liu et al., 2017). Despite a certain level of automation achieved in recent decades, obtaining AST results in modern clinical laboratories still requires more than 10 h following bacterial isolation (Van Belkum and Dunne, 2013). As a result, one to 3 days of delay occur between the initiation of empirical antimicrobial therapy and the AST result. Therefore, a stand-alone AST method would be ideal to reduce the time to result. From a basic microbiological perspective, a stand-alone AST method needs to address the challenges of unknown pathogen identity and microbial load and be able to adapt to the fast-changing understanding of current antibiotic resistance mechanisms, the discovery of new mechanisms, epidemiological aspects, variation of the growth-associated lag time, and heterogeneity of resistance. The presented method aims to address these challenges by utilizing a biosensor to perform AST directly from unprocessed clinical specimens. Biosensors can be categorized by the reactant that they measure after the recognition event. Specifically, biosensor types include optical (light), bioluminescent (photons), thermal (heat), mass (resonance frequency changes), and electrochemical (electron transfer). While each of these types has inherent strengths and weaknesses, optical and electrochemical biosensors have become the most widely used. Although optical biosensors have been adopted throughout clinical diagnostics and life science research due to their speed and sensitivity, most still require the use of target amplification such that the signal is enhanced to a measurable level. Of these amplification methods, real-time PCR has emerged as the most widely adopted and is now considered the gold standard for the detection of nucleic acids from a variety of origins. Our method utilizes electrochemical biosensors due to their superior speed and selectivity at a low cost. Historically, however, electrochemical biosensors have lacked sufficient sensitivity for use beyond glucose monitoring and clinical chemistry analysis. Unlike previously reported electrochemical sensors that used graphite or carbon electrodes, a single layer of gold (Au) is used in this method for all three electrodes, i.e., working, auxiliary, and reference electrodes. Typically, Ag/AgCl or a saturated calomel electrode (SCE) is used as the reference electrode so that reversible oxidation/reduction occurs at a fixed potential at the reference electrode. In contrast, Au is used as the reference electrode in this method because its malleability and durability simplify fabrication and allow for extremely thin electrodes. In this particular application, where the reduction of a mediator is monitored, Au can be successfully used as the reference electrode because a low voltage difference is maintained for short periods of time. The Au/Au/Au electrode system is characterized by cycling enzymatic amplification and targets are measured by amperometry (Gau et al., 2005). The high turnover number of mediated HRP-TMB redox-cycling amplification replaces the need for nucleic acid amplification, and the antifouling properties of the casein coating on all electrodes enable direct-from-specimen detection with a baseline level that is less than 50 nA (Liao et al., 2007; Pan et al., 2010).

The major challenges of conventional electrochemical-based biosensors are lack of detection sensitivity and interference with matrix components from unprocessed specimens, therefore most electrochemical detection methods only report qualitative measurement of high abundant analytes, not quantification of extremely low abundant analytes (<10^5^ CFU/ml for urine and <10 CFU/ml for blood) directly from specimens. However, in the presented study, we were able to detect as low as one single colony in 0.5 ml of whole blood (Figure 5) by removing matrix interferents at the start of the assay. Furthermore, we were able to perform categorical susceptibility and MIC reporting of clinical isolates in Figures 6, 7 by generating microbial response curves of electrochemical signal levels in nano amperes (nA) with an elaborated one-drug stripwell. With this method, we then established a dual-dilution signature for direct-from-urine AST and a library of response curves at different microbial loads in Figure 8. The feasibility of the direct-from-specimen AST with unknown microbial loads, susceptibility, or species was demonstrated with blinded contrived urine samples with S, I, and R susceptibility to gentamicin, as well as a polymicrobial sample consisting of microorganisms with different susceptibilities in Figure 9.

The LoD is the lowest concentration at which analyte can be detected 95% of the time (a 5% likelihood of a false negative). Alternatively stated, the LoD is the true value where the likelihood of a false negative measurement is 5%. The much smaller blood volume (200–500 µL) required for point-of-care or pediatric applications introduces a new dilemma of a compromised LoD. Even with the successful identification of just 1 bacterium in 500 µL of whole blood, the best LoD is 6 CFU/ml, which seems to be inferior to the 1 CFU/ml LoD criteria for adult sepsis with a 10-ml blood sample. In the preliminary evaluation of detecting whole blood contrived at 2 CFU/ml, only 4 out of 24 (16.7%) 0.5-ml samples were reported positive above the limit of blank (LoB) of 50 nA from the electrochemical current quantification. While most 2 CFU/ml samples were reported negative, the signal levels of the positive samples suggested one sample each harbored 1 or 2 colonies. One goal of this study was to achieve the best clinical sensitivity and specificity to identify all blood borne pathogens in 200–500 µL blood volume in less than 8 h. Uropathogens usually appear in high abundance (>10^8^ CFU/ml), so the total assay time for direct-from-urine pathogen ID can be as short as 2 h (data not shown).

The removal of interfering matrix components in blood or urine and a comparison of microbial responses from different drug-to-bug ratios can enable antimicrobial susceptibility reporting in hours directly from unprocessed specimens instead of days from clinical isolates as demonstrated in Figures 8, 9. The presented direct-AST reports Resistant (R), Intermediate (I), and Susceptible (S) results correlated to CLSI breakpoints and MICs determined by CLSI methodology used in traditional AST. In Figures 8, 9, we established a library of microbiological signatures from a range of microbial loads with an electrochemical-based molecular analysis platform and demonstrated robust performance when validated in three representative susceptibility categories covering R, I, and S. The detection method described utilizes dual-dilution kinetic curves and automatable molecular quantification of species-specific 16S rRNA through the use of an electrochemical sensor to assess microbiological responses to antibiotic exposure.

**Limitations and Future Plan**–We have demonstrated the feasibility of a direct-ID/AST assay, which we plan to further optimize and validate. The presented study included a select group of antibiotics, species, and susceptibilities; therefore, we will expand the antibiotic panel, target species, and polymicrobial conditions. We will also test different contrived concentrations of representative pathogens to match the microbial loads and polymicrobial combinations in adult blood and urine for evaluating microbial recovery rate. Additionally, we tested only Gram-negative bacteria due to the lack of an important outer membrane layer in Gram-positive bacteria that is present in Gram-negative bacteria, making Gram-positive organisms less resistant to most antibiotics than Gram-negative ones (Breijyeh et al., 2020). Therefore, we plan to scale up the species-specific probe pairs for both Gram-positive and Gram-negative bacteria to cover clinically relevant organisms and antibiotic selections for each infection site and increase the clinical utility and commercial value. Finally, the presented method is intended to be automated to reduce the workload and hands-on time for ID and AST. Once automated, a future study with a much larger sample size of prospectively collected specimens in a clinical trial is needed to cover the most common causative pathogens in blood and urine specimens found in clinical settings.

## Data Availability

The original contributions presented in the study are included in the article/Supplementary Material, further inquiries can be directed to the corresponding author.
