# Supplementary material for: Rapid Electrochemical-Based PCR-Less Microbial Quantification and Antimicrobial Susceptibility Profiling Directly From Blood and Urine With Unknown Microbial Load or Species
Source: Front Bioeng Biotechnol. 2021 Sep 16;9:744198. doi: 10.3389/fbioe.2021.744198 (PMC8481646; doi:10.3389/fbioe.2021.744198)
Supplement: Supplementary file 1 [file DataSheet2.PDF]

**Supplemental Table 2.** Data table for Figure 9 blinded samples.

| **Figure 9A. $10^8$ CFU/mL** | | | | | | | | |
|---|---|---|---|---|---|---|---|---|
| Sample | GC Signal (nA) | $2.50 \times 10^7$ Ratio | $1.25 \times 10^7$ Ratio | $6.25 \times 10^6$ Ratio | $2.94 \times 10^6$ Ratio | $1.47 \times 10^6$ Ratio | $7.35 \times 10^5$ Ratio | $3.68 \times 10^5$ Ratio |
| EC61 (R) | 9919.1 | 1.00816 | 1.00816 | 1.00816 | 0.99546 | 1.00816 | 0.90151 | 0.17149 |
| **Figure 9B. $10^7$ CFU/mL** | | | | | | | | |
| Sample | GC Signal (nA) | $2.50 \times 10^6$ Ratio | $1.25 \times 10^6$ Ratio | $6.25 \times 10^5$ Ratio | $2.94 \times 10^5$ Ratio | $1.47 \times 10^5$ Ratio | $7.35 \times 10^4$ Ratio | $3.68 \times 10^4$ Ratio |
| EC114 (I) | 9526.61 | 0.53039 | 0.20639 | 0.17077 | 0.05904 | 0.01416 | 0.01063 | 0.00844 |
| **Figure 9C. $10^6$ CFU/mL** | | | | | | | | |
| Sample | GC Signal (nA) | $2.50 \times 10^5$ Ratio | $1.25 \times 10^5$ Ratio | $6.25 \times 10^4$ Ratio | $2.94 \times 10^4$ Ratio | $1.47 \times 10^4$ Ratio | $7.35 \times 10^3$ Ratio | $3.68 \times 10^3$ Ratio |
| EC67 (S) | 2853.51 | 0.09104 | 0.06342 | 0.05839 | 0.01337 | 0.01347 | 0.01049 | 0.00875 |
| **Figure 9D. $10^5$ CFU/mL** | | | | | | | | |
| Sample | GC Signal (nA) | $2.50 \times 10^4$ Ratio | $1.25 \times 10^4$ Ratio | $6.25 \times 10^3$ Ratio | $2.94 \times 10^3$ Ratio | $1.47 \times 10^3$ Ratio | $7.35 \times 10^2$ Ratio | $3.68 \times 10^2$ Ratio |
| EC67(S) + EbC154(R) | 158.134 | 1.0746 | 1.01882 | 1.04135 | 0.20976 | 0.19721 | 0.22629 | 0.20424 |
