# Supplementary material for: Rapid Electrochemical-Based PCR-Less Microbial Quantification and Antimicrobial Susceptibility Profiling Directly From Blood and Urine With Unknown Microbial Load or Species
Source: Front Bioeng Biotechnol. 2021 Sep 16;9:744198. doi: 10.3389/fbioe.2021.744198 (PMC8481646; doi:10.3389/fbioe.2021.744198)
Supplement: Supplementary file 2 [file DataSheet1.pdf]

Supplemental Table 1. Data table for Figure 8 dual dilution response curve library.

**Figure 8A. 10^8 CFU/mL**								
Sample	GC Signal (nA)	$2.50x10^7$ Ratio	$1.25x10^7$ Ratio	$6.25x10^6$ Ratio	$2.94x10^6$ Ratio	$1.47x10^6$ Ratio	$7.35x10^5$ Ratio	$3.68x10^5$ Ratio
EC67 (S)	10143.4	0.84114	0.49463	0.4047	0.04738	0.03195	0.02503	0.0289
KP39 (I)	10000	1	1	1	0.97502	0.84697	0.47504	0.18299
EC11 (R)	9641.82	0.96217	1.01837	1.00257	0.884933	0.90528	0.88932	0.82178
**Figure 8B. 10^7 CFU/mL**								
Sample	GC Signal (nA)	$2.50x10^6$ Ratio	$1.25x10^6$ Ratio	$6.25x10^5$ Ratio	$2.94x10^5$ Ratio	$1.47x10^5$ Ratio	$7.35x10^4$ Ratio	$3.68x10^4$ Ratio
EC67 (S)	10240	0.09592	0.05414	0.03203	0.00691	0.0065	0.00653	0.00853
KP39 (I)	3653.12	2.00059	2.35434	1.51172	0.6445	0.36067	0.10841	0.0496
EC11 (R)	10000	1	1	0.76496	0.4359	0.34206	0.31141	0.07408
**Figure 8C. 10^6 CFU/mL**								
Sample	GC Signal (nA)	$2.50x10^5$ Ratio	$1.25x10^5$ Ratio	$6.25x10^4$ Ratio	$2.94x10^4$ Ratio	$1.47x10^4$ Ratio	$7.35x10^3$ Ratio	$3.68x10^3$ Ratio
EC67 (S)	5528.37	0.01863	0.01122	0.00921	0.00681	0.00568	0.00585	0.0054
KP39 (I)	2717.95	0.83489	0.52083	0.19908	0.09294	0.05265	0.02571	0.02142
EC11 (R)	2192.72	1.70512	1.96059	0.71589	0.24497	0.19567	0.166	0.05074
**Figure 8D. 10^5 CFU/mL**								
Sample	GC Signal (nA)	$2.50x10^4$ Ratio	$1.25x10^4$ Ratio	$6.25x10^3$ Ratio	$2.94x10^3$ Ratio	$1.47x10^3$ Ratio	$7.35x10^2$ Ratio	$3.68x10^2$ Ratio
EC67 (S)	266.72	0.13651	0.08554	0.11308	0.10076	0.14294	0.1406	0.13942
KP39 (I)	373.788	0.93524	0.4028	0.2311	0.13792	0.1014	0.09279	0.09276
EC11 (R)	283.539	1.06794	1.06075	0.65143	0.16841	0.1964	0.16585	0.11027
